# ROC curve analysis: a useful statistic multi-tool in the research of nephrology

**DOI:** 10.1007/s11255-024-04022-8

**Published:** 2024-03-26

**Authors:** Stefanos Roumeliotis, Juul Schurgers, Dimitrios G. Tsalikakis, Graziella D’Arrigo, Mercedes Gori, Annalisa Pitino, Daniela Leonardis, Giovanni Tripepi, Vassilios Liakopoulos

**Affiliations:** 1 2nd Department of Nephrology, Medical School, AHEPA Hospital, Aristotle University of Thessaloniki, 54636 Thessaloniki, Greece; 2 Department of Electrical and Computer Engineering, University of Western Macedonia, Kozani, Greece; 3 Institute of Clinical Physiology (IFC), National Research Council (CNR), 89124 Reggio Calabria, Italy; 4 Institute of Clinical Physiology (IFC), National Research Council (CNR), 00100 Rome, Italy

**Keywords:** Area under the curve, Diagnostic test,, Discriminatory ability, Receiver operator characteristic curve, Sensitivity, Specificity

## Abstract

In the past decade, scientific research in the area of Nephrology has focused on evaluating the clinical utility and performance of various biomarkers for diagnosis, risk stratification and prognosis. Before implementing a biomarker in everyday clinical practice for screening a specific disease context, specific statistic measures are necessary to evaluate the diagnostic accuracy and performance of this biomarker. Receiver Operating Characteristic (ROC) Curve analysis is an important statistical method used to estimate the discriminatory performance of a novel diagnostic test, identify the optimal cut-off value for a test that maximizes sensitivity and specificity, and evaluate the predictive value of a certain biomarker or risk, prediction score. Herein, through practical examples, we aim to present a simple methodological approach to explain in detail the principles and applications of ROC curve analysis in the field of nephrology pertaining diagnosis and prognosis.

## Introduction

During the past decade, scientific research in the area of Nephrology has focused on evaluating the clinical utility and performance of various biomarkers for diagnosis, risk stratification and prognosis. Before suggesting the use of a biomarker in everyday clinical practice to identify a specific disease or condition (for example troponin for the diagnosis of acute myocardial infarction), specific statistic measures should evaluate the diagnostic accuracy and performance of this marker. The first, fundamental tests used to statistically evaluate the diagnostic performance of a new marker are sensitivity and specificity. Sensitivity or true positive rate, determines the proportion of diseased subjects with positive test result for the new marker, whereas specificity or true negative rate, determines the proportion of disease-free subjects with a negative test result. Thus, sensitivity (defined as the ratio of true positives to the sum of false negatives and true positives) evaluates the ability of the test/marker to correctly identify patients who actually have a certain disease or condition. On the other hand, specificity (defined as the ratio of true negatives to the sum of false positives and true negatives) tests the capacity of this marker to correctly classify patients as disease-free. Accuracy is a measure of overall correctness of a diagnostic test, represents the proportion of correctly classified cases (both true positives and true negatives), calculated by dividing the sum of true positive and true negative by the sum of all cases. Two additional mathematical tests, positive predictive value (the proportion of truly positives among all positive results) and negative predictive value (the proportion of truly negatives among all negative results), provide answers to the clinically relevant question of whether an individual will be correctly diagnosed as having the disease according to the test/marker result (Table 1).

**Table 1:** Diagnostic value indices of a test or biomarker

Statistic measures of a test or biomarker	
Accuracy	(True positives + true negatives)/(true positives + true negatives + false positives + false negatives)
Sensitivity	True positives/(true positives + false negatives)
Specificity	True negatives/(true negatives + false positives)
Positive predictive value	True positives/(true positives + false positives)
Negative predictive value	True negatives/(true negatives + false negatives)

**Fig. 1:**
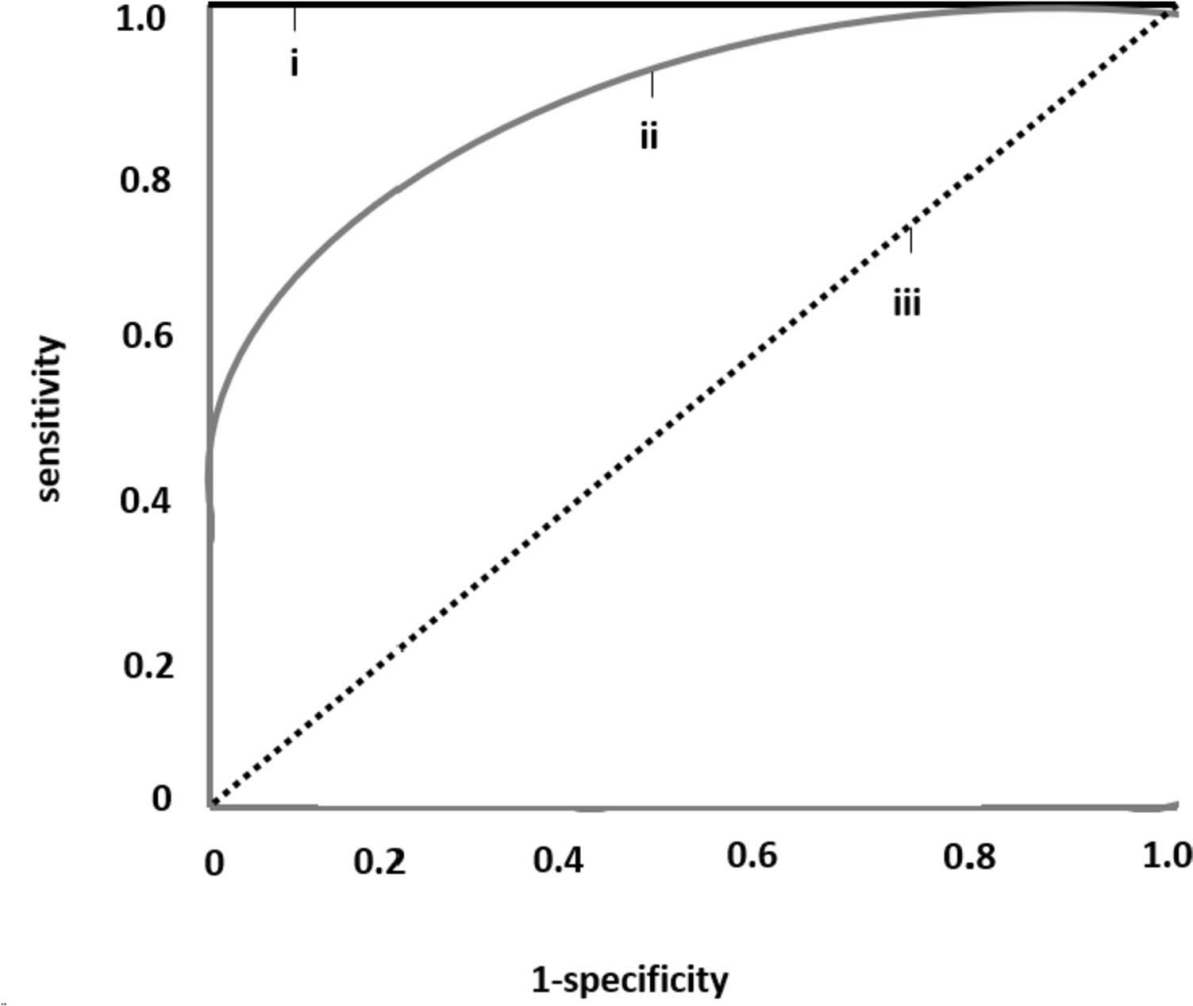
Receiver operating characteristics (ROC) curves. Diagonal, dotted, black line (iii): a diagnostic test with the lowest discriminatory ability, which is no better than chance, area under the curve (AUC) = 0.5. The value of AUC is accompanied by 95% CIs. Gray line (ii): a test with a modest discriminatory ability of about 0.5. Black line (i): a perfect, accurate diagnostic test (highest sensitivity (100%) and specificity (100%)), AUC = 1

Therefore, a novel, proposed diagnostic test should have high discriminatory power to accurately classify all tested subjects as healthy or diseased. However, the test or biomarker available often consists of a continuous variable and there is a need to identify a cut-off threshold capable of discriminating between healthy and diseased subjects. A recent example that everyone might be familiar with, is the self-administered, rapid tests for COVID-19 antigen: two red lines (one in the Control region and one in the Test region), regardless of intensity, indicate a positive result, whereas a sole red line in the Control region and no colored line in the Test region indicates a negative result. These diagnostic tests are dichotomous, as they provide a “yes” or “no” answer regarding whether a subject has the disease or not. However, the biomarkers on which these tests are based on, such asCOVID-19 antigen, are quantitative, expressed in continuous terms and need to be transformed into a dichotomous variable. The Receiver Operating Characteristic (ROC) Curve is a statistic method used to assess the discriminatory ability of a quantitative marker across the whole range of all its values, when subjects are correctly categorized as diseased and non-diseased (or with and without an incident event) by a gold standard, reference test [1–3]. A typical ROC curve is shown in Fig. 1, where the x-axis represents the false positive rate, defined as 1 minus specificity and the y-axis represents the sensitivity (true positive rate). The ROC curve of a given test/biomarker is built-up by specific algorithms implemented in most statistical software. The algorithms calculate, for a series of thresholds of the variable being tested, sensitivity (i.e., the true positive rate) and 1-specificity (i.e., the false positive rate). True positives (Y scale) and false positives (X scale) as derived by the procedure are reported in a cartesian graph and the conjunction of the coordinates generated by the various thresholds provides a ROC curve. The dotted diagonal line (iii) represents a diagnostic test with the lowest possible discriminatory power, similar to chance, with an AUC of 0.5, whereas the black line (i) is the perfect test with 100% sensitivity and specificity with an AUC of 1.0. The gray line (ii) is a typical good curve, with an accuracy of approximately 80%. For instance, consider that for each threshold of the variable being tested the true and false positive rates be identical. As a consequence, the area under the ROC curve will be 50% (see dotted diagonal line in the graph-iii), implying that the variable is absolutely useless for identifying the condition of interest. Otherwise, if for each threshold of the variable being tested the true positive rate is 100% and the false positive rate is zero, the consequence is that the area under the ROC curve will be 100% (see the black line in the graph-i) implying that the variable is absolutely accurate for identifying the condition of interest. The area under the curve (AUC) of the ROC curve represents the discriminatory power of the curve/test, with values ranging from 0.5 (lowest) to 1.0 (highest and most accurate) and 95% Confidence Intervals. This is a range of values that provides an estimate of the uncertainty associated with the true underlying ROC curve and helps to assess the reliability of the ROC curve analysis and the performance of a classification model in distinguishing between classes. Typically, it is computed using methods like bootstrapping or resampling techniques.

Ideally, after ROC curve analysis investigates the discriminatory power of a novel diagnostic test (compared to the gold, standard reference test), these results should be validated in another (external) population, to avoid under- or overfitting of the statistical model on which the test was based.

Historically, ROC analysis was first used during World War II to assist radar operators in deciding whether a blip on the radar screen corresponded to a sound or a moving object and it was later adopted by diagnostic statistic research. During recent years, ROC curves have been used quite broadly. A quick search in the Medline database shows that over the past decade, the use of ROC curves in clinical nephrology research has significantly increased. Using the search key words “ROC curve” AND “CKD” OR “ESKD” OR “Hemodialysis” OR “Peritoneal Dialysis” in the abstract or title generated 307 papers in the decade 2003–2013, a number that quadrupled in the past decade (2013–2023) to 1332 papers. This increase might be attributed to an exponential growth of clinical and epidemiological research evaluating the potential accuracy or predictive ability of various biomarkers in the field of nephrology in these recent years. The applications of ROC curve analysis include the estimation of discriminatory performance of a novel diagnostic test, the identification of the optimal cut-off value for this test that maximizes sensitivity and specificity, the assessment of the predictive value of a certain biomarker, and the evaluation of multivariate risk scores based on multiple variables or risk factors. Herein, through clinical practical examples, we aim to present a simple methodological approach explaining in detail all the principles and applications of ROC curve analysis in the field of nephrology.

## Examples

### Example 1: Using ROC curve to evaluate the discriminatory performance of a novel diagnostic tool

**Fig. 2:**
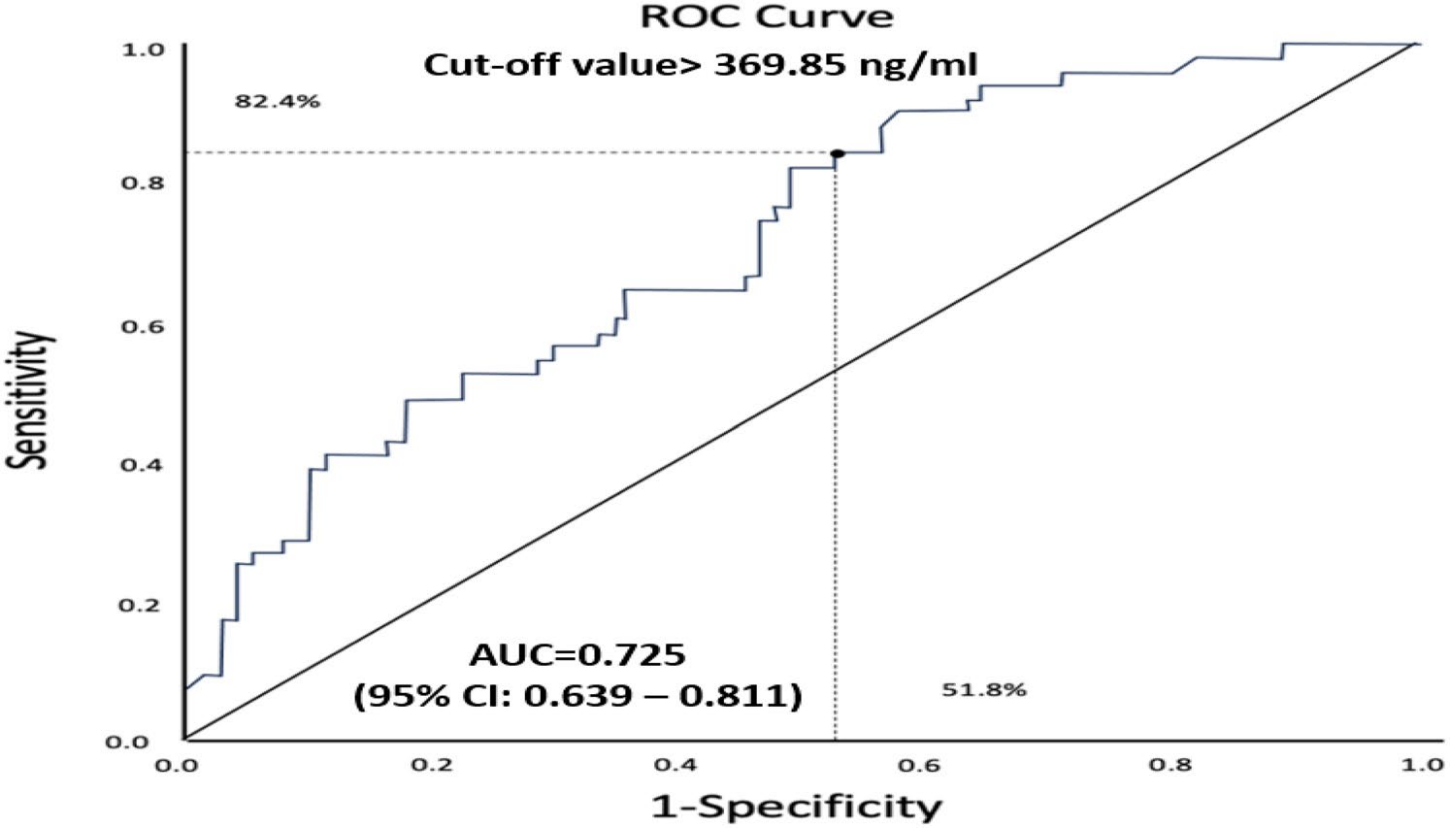
ROC curve showing optimal cut-off value for serum asprosin, with an AUC of 72.5% for cutoff > 369.85 ng/ml (black dot), dotted black lines show sensitivity and specificity for this specific cut-off value, 82.4% and 51.8% respectively

ROC curve analysis can be used to evaluate if a novel biomarker might be useful in the diagnosis of a certain condition or disease. This was done by Zhou et al. [4],to investigate the possible association between a new biomarker, asprosin and metabolic syndrome (MS) in a cross-sectional study enrolling134 hemodialysis (HD) patients. According to the definition by the International Diabetes Federation (gold standard), MS was diagnosed in 51 patients. First, the authors found that, HD patients with MS had significantly higher circulating levels of asprosin (371.5 ± 144.9 ng/ml and 502.2 ± 153.3 ng/ml, respectively, Mann–Whitney test), compared to patients without MS. Second, regression analysis showed that asprosin was independently associated with MS with an odds ratio of 1.008, after adjustment for several well-known risk factors. Third, the authors explored whether asprosin could predict MS, through a ROC curve analysis. They found an AUC of 72.5% with 95% confidence interval-CI of 63.9 – 81.1 (Fig. 2). Another characteristic of the ROC curve, besides AUC is the CI; in this case, narrower CIs (i.e., a smaller range between minimum and maximum values) corresponds to a better and stronger discriminatory ability of the diagnostic test. Finally, based on the ROC curve, the authors determined the optimal cut-off value of asprosin for identifying MS, which was set at 369.85 ng/mL This cut-off point is provided by the best combination of sensitivity and specificity, which were82.4% and 51.8%, respectively (Fig. 2). It is clear that, although the first analyses (Mann–Whitney test and multiple regression analysis) showed a possible association between asprosin levels and MS, the ROC curve indicates that asprosin was not such a good marker for predicting MS, given its low specificity, relatively modest sensitivity and broad CIs.

### Example 2: Using ROC curve to evaluate the prognostic role of a biomarker and to determine the optimal cut-off value

**Fig. 3:**
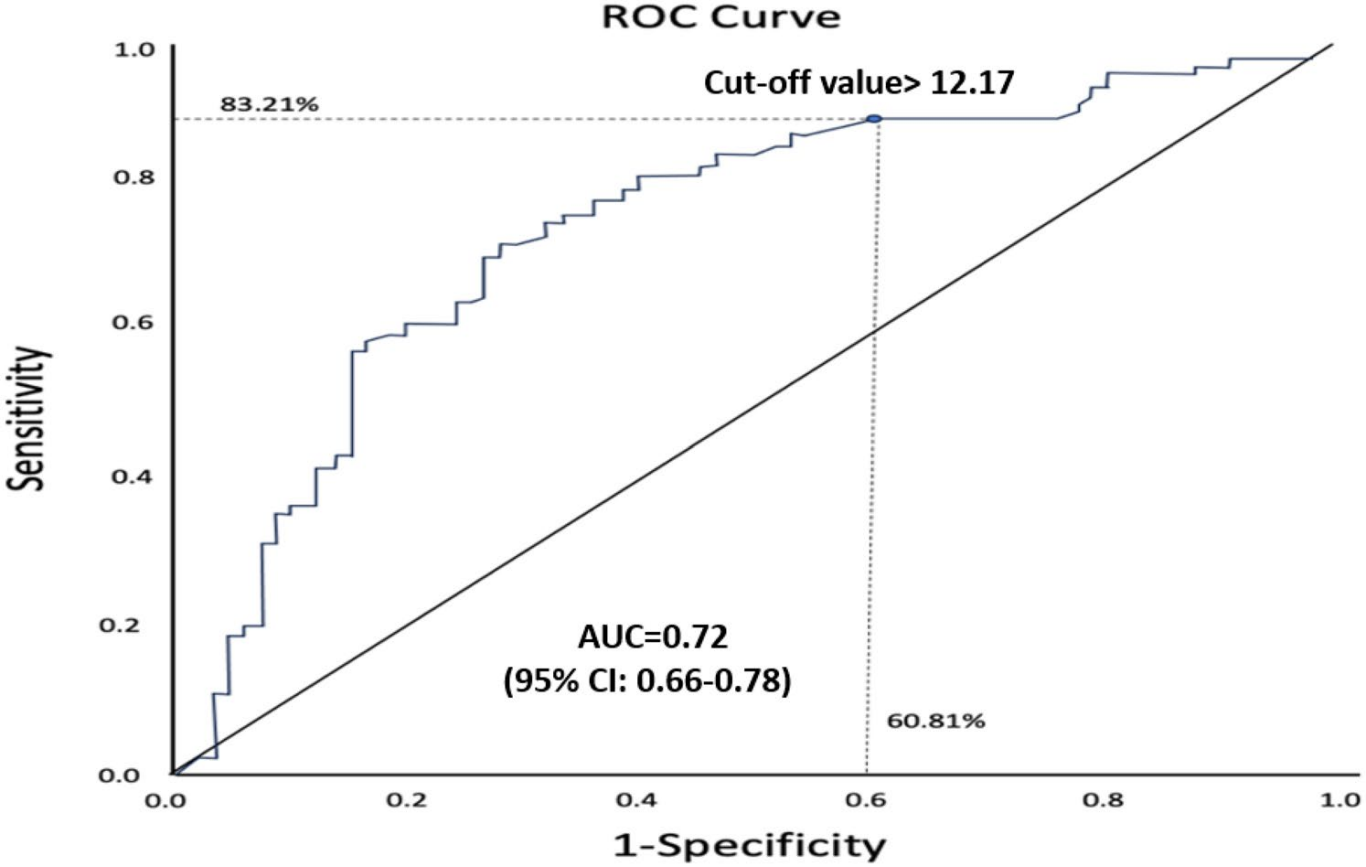
ROC curve showing optimal cut-off value for UAR, with an AUC of 72% for cutoff > 12.17(black dot). dotted black lines show sensitivity and specificity for this specific cut-off value, 83.21% and 60.81%, respectively

The urea-to-albumin ratio (UAR) is a novel predictor of adverse events —including mortality— in septic patients hospitalized in the intensive care unit (ICU). Recently, Rodrigues et al. [5] investigated the potential predictive value of UAR for mortality in critically ill COVID-19 patients. They conducted a retrospective study, enrolling 211 high-risk patients admitted with COVID-19 infection in the ICU. As expected, the mortality rate in this cohort was high (64.9%). The authors aimed to investigate the classification accuracy of this marker for ICU mortality using a ROC curve analysis. Thus, the first step consisted of determining the disease status for all patients, using a gold standard method, i.e., distinguishing between survivors and non-survivors. To evaluate the discriminatory ability of UAR for predicting ICU mortality in COVID-19 patients, the authors performed a ROC curve analysis, across all paired values of sensitivity with 1 minus specificity (i.e., the false positive rate), Fig. 3. The authors then proceeded with determining the optimal cut-off value of UAR to predict this outcome. This value can be identified by the ROC curve and should ensure the highest specificity and sensitivity combination possible. However, as clearly shown in Fig. 3, a trade-off exists between sensitivity and specificity, where the choice of a higher sensitivity is inevitably accompanied by a lower specificity.

Since AUC measures the entire two-dimensional area underneath the entire ROC curve, in this example (Fig. 3), the AUC derived from the ROC curve is 0.72 with 95% CI: 0.66–0.78). Moreover, in this example, the optimal threshold of UAR to predict ICU mortality was found to be above 12.17, which corresponded to a 83.21% sensitivity and a specificity of 60.81%. A sensitivity of 83.21% implies that out of 100 individuals who will die, 83 would be correctly classified as non-survivors. However, this also means that among these 100 patients, 17 would not have a positive test result (in this example, 16.79% of patients that died had UAR below 12.17). A specificity of 60.81% indicates that out of 100 patients who will survive, 61 would be correctly classified as survivors by the test and 39 incorrectly classified as non-survivors (in this example, 39.20% of survivors had increased UAR above 12.17).

Subsequently, the authors computed all the indices of using the UAR threshold of 12.17 to identify survivors in patients hospitalized in ICU due to COVID-19 infection:

–sensitivity = 83.21;
–specificity = 60.81;
–false positive rates (1-specificity) = 39.19;
–positive predictive value (PPV) = 79.72;
–negative predictive value (NPV) = 66.18% and;
–accuracy = 75.36% in this example.

Τhe ROC curve combines sensitivity (y-axis) and 1 minus specificity (x-axis), representing all possible cut-off values the test might have. Typically, a ROC with an AUC above 75% is considered to be clinically useful. However, determining the cut-off value usually depends on the goal of the test, due to the trade-off between specificity and sensitivity. The authors reported that compared to survivors, those who died had increased UAR, with a mean difference of 12.8. Additionally, Cox regression analysis (the final step of their analysis) showed that a UAR value above 12.17 doubled the risk of all-cause mortality in this cohort. In this example, ROC curve analysis was used to assess the prognostic value of a biomarker and to determine the optimal cut-off value.

### Example 3: Using ROC curve to develop and validate risk prediction models or scores

ROC curve can also be used to develop and validate novel risk prediction models or scores. For instance, You et al. [6], aimed to construct a risk prediction model for CV disease in HD patients and conducted a prospective study in 388 maintenance HD, followed for a mean of 3.27 years with the occurrence of CV events as endpoint. During the follow-up period, 132 patients had a CV event. To build the new prediction model, the first step was to identify risk factors for the outcome. Among the 26 candidate prognostic variables that were tested, stepwise Cox regression analysis revealed that hypertension, diabetes mellitus, age ≥ 65 years and abnormal white blood cell count were the sole independent predictors. Next, the authors developed a simple risk prediction score by assigning1 point to each of these 4 risk factors. Compared to patients with none of these factors, patients with 1,2, and over 3 factors had significantly, graded risk for CV disease (HRs: 3.29, 7.42, and 15.43, respectively). The subsequent step was to evaluate and validate the calibration of this risk score, by comparing the difference between predicted and actual risk with the ROC curve analysis. To increase the validity of their analysis, the authors initially performed this risk analysis first in their population and then applied it to a bootstrap validation data set. In both sets, this risk prediction score showed acceptable discriminatory performance for CV disease (AUC = 0.70, 95% CI 0.65–0.75 and AUC = 0.69, 95% CI 0.66–0.72, respectively). The authors concluded that given the high prevalence of CV disease in this high-risk population, a simple CV risk model based on variables that are easily obtained with little cost could be of clinically useful.

### Example 4: Using ROC curve to compare two risk prediction models and to determine their relative performance

**Fig. 4:**
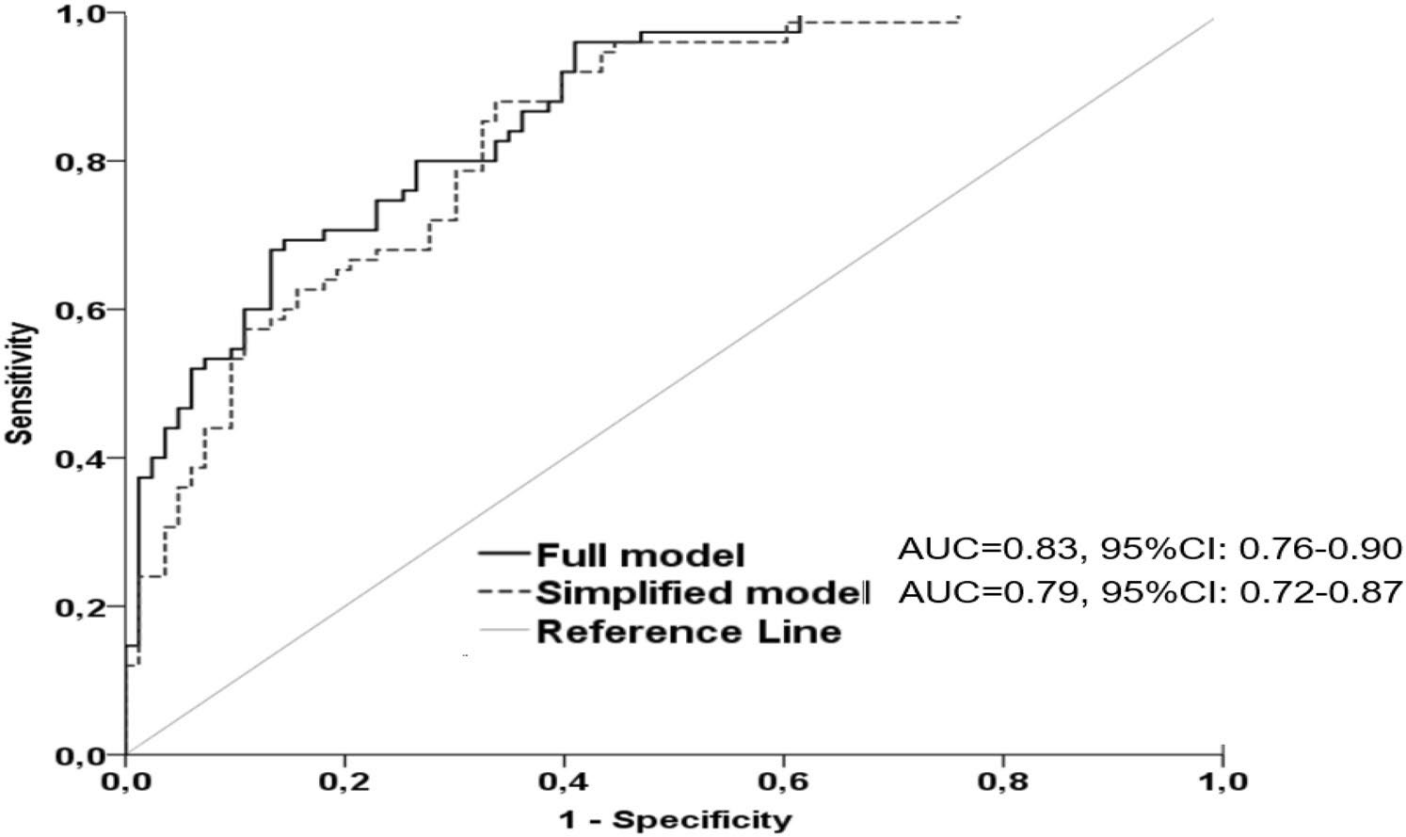
*ROC* curves comparing the discriminatory performance of the full and simplified risk model in predicting *CV* disease in patients with diabetic kidney disease

Another application of the ROC curve analysis is the comparison of two risk prediction models, to determine their relative performance and identify the one with superior predictive ability, thus enabling a quantitative evaluation of their overall accuracy. In a recent study conducted by our team, we aimed to find a risk predictive model that was simple, quick, easy to evaluate and accurate for assessing the CV risk in subjects with diabetic kidney disease [7]. One hundred fifty-eight patients with different degrees of renal function and type II diabetes for at least 10 years were enrolled. At baseline, various demographic, clinical, anthropometric, and biochemical variables were collected. Moreover, carotid intima-media thickness (cIMT) was evaluated by ultrasound as a surrogate marker of subclinical atherosclerosis. All patients were followed for a long period of 7 years, with fatal or nonfatal CV events as the primary endpoint (75 events). To assess the predictive value of various variables collected at baseline, non-CV death was considered as a competitive event, using Fine-Gray regression models. Survival analyses revealed that among all variables, male gender, history of CV disease, long duration of diabetes, low hemoglobin, low estimated glomerular filtration rate (eGFR), high albuminuria, low high density lipoprotein (HDL) cholesterol, low serum albumin and high cIMT were independently associated with the CV outcome. Then, in multivariate models, only history of CV disease, eGFR and albuminuria remained significantly associated with the outcome of interest. Next, a risk model was developed with all these nine variables. However, the assessment of all these data are time consuming, expensive and laborious. To address the clinical question regarding the clinical utility of cIMT measurement and simplify the existing full, 9-variable model, a simpler (nested) risk model with only three variables (eGFR, albuminuria and history of CV disease) was constructed. The performance of these two models, was compared with various statistical tests, with different purposes. First, a log likelihood test demonstrated that there was no significant difference between the data fitting of these two models (*x*^2^ = 9.48, 6 df, *p* = 0.15). Second, Hosmer–Lemeshow test revealed that the simplified model was better calibrated compared to the full model (respectively, *x*^2^ = 9.24, *p* = 0.32 and *x*^2^ = 11.09, *p* = 0.20).). Finally, the discrimination performance of these two models was compared using the ROC curve analysis (Fig. 4).

This test confirmed that these two risk models had nearly identical and high accuracy to predict CV events [full model: AUC 0.87 95% CI: 0.81–0.92 and simplified model: AUC 0.84, 95% CI: 0.78–0.90 respectively]. Based on these analyses, we concluded that the simple risk model consisting of three variables that are easy to measure, quick and cheap might be used to predict CV events in diabetic CKD. The time-consuming and elaborate diagnostic tests such as the cIMT measurement do not actually offer much in the risk assessment. However, for these results to be adopted in everyday clinical practice, they should be validated in different, external, large-scale cohort studies.

### Example 5: Using ROC curve analysis to reevaluate the epidemiology of a disease in a specific population

Another, innovative use for the ROC curve analysis is to reevaluate the epidemiology of a certain disease in a specific population. Long-standing epidemiologic evidence suggest that increased triglycerides (TGs) are associated with CV morbidity and mortality. However, the exact value at which risk starts to increase has yet not been identified. This topic has been investigated in a large, multicenter, national, population-based cohort, the URRAH study [8], which included 14,189 subjects followed for a long period of 11.2 years with outcome the incidence of CV events. The authors performed a ROC curve analysis to find the optimal, early, prognostic cut-off of TGs for predicting CV events, using the incidence of CV events as the dichotomous (yes or no) classification variable and TGs as the basic variable. They computed the pairs of sensitivity–specificity for all the range of TGs values. The ideal cut-off value was determined by Youden index test, which identifies the threshold value corresponding to the point of the curve nearest the upper-left corner (corresponding to 100% specificity and 100% sensitivity), as described before [3], with the following equation: J = max (sensitivity + specificity –1). From the ROC curve, the optimal cut-off value of TGs to predict CV events was found to be 89 mg/dl, which had a sensitivity of 76.6%, a specificity of 34.1% and an AUC of. 0.569 (95% CI 0.561–0.578). It should be noted that the CIs are quite narrow, indicating a “solid” discriminatory performance and the cut-off value identified had the maximum sum of sensitivity + specificty. This optimal cut-off value, of 89 mg/dL, is lower than the conventionally used cut-off value of 150 mg/dL (sensitivity 33.0% and specificity 74.3%). The next step of the statistical analysis in this paper was to separately insert, both the prognostic (> 89 mg/dl, as found by theROC curve) and the standard, conventional 150 mg/dl threshold values as independent variables in multivariate Cox models (adjusted for well-established CV risk confounders), with CV events as the dichotomous, dependent variable. Based on the HRs (conventional = 1.211, 95% CI 1.063–1.378, prognostic = 1.15, 95% CI 1.021–1.295), both cut-off values were considered acceptable, independent predictors of CV events in the whole cohort. Therefore, the authors concluded that significantly lower (61 mg/dl lower than standard) cut-off level of TGs predicts CV disease and therefore, these subjects should be carefully monitored in primary care. Although in this example, the two different thresholds (89 vs 150 mg/dL) were independent predictors of CV events, it should be noted that they have different sensitivity and specificity, with the conventionally used threshold (150 mg/dL) having a low sensitivity (33%) and high specificity (74%), and the new threshold proposed by the researchers (89 mg/dL) having a high sensitivity (77%) and a low specificity (34%). This should be taken under consideration if the new threshold was used for monitoring patients in the primary care, because not only more patients who will have the event would be actually identified (and the events prevented) but also there would be more false negatives, implying more frequent exams and visits for patients who will not eventually develop the CV event. Therefore, the “best” threshold for a test, depends on the test’s goal from a clinical and practical perspective.

## Conclusion

ROC curve analysis is an important and widely used statistical test with various applications in the research field of nephrology. This statistical method evaluates the diagnostic performance of a novel test or marker, assesses the predictive ability of a marker, identifies the ideal cut-off values of a test and allows the comparison of the diagnostic performance between two or more risk prediction models. Since this current decade is the era of biomarkers and predictive tests in nephrology, ROC analysis is an essential and easy tool to validate the actual clinical utility of proposed markers and tests. Measures like reclassification, calibration statistic, net reclassification index, and integrated discrimination improvement may not be as widely adopted or as easily interpretable as ROC curves, limiting their utility in certain contexts in clinical research. However, in specific scenarios or when combined with ROC analysis, they can provide complementary information for a more thorough assessment of model performance. The main advantages of ROC curves are the following: (1) this analysis evaluates the performance of a test/biomarker across all possible thresholds, providing insights into the overall discriminatory power of the biomarker of interest without being dependent on a specific threshold; (2) ROC curves illustrate the trade-off between true positives and false positives, allowing for a visual representation of how well a biomarker discriminates across various threshold values; (3) the AUC derived from ROC curves provides a single, interpretable summary of a model’s discriminatory power. Higher AUC values indicate better overall performance, making it easy to compare models and determine their relative effectiveness.

Although, ROC curve has several important applications in research and clinical practice, there are also certain limitations and pitfalls that should be taken under consideration. First, it provides a graphical representation of the diagnostic accuracy of the test across all possible thresholds, but does not directly indicate the optimal threshold for making decisions in practical applications. Second, while ROC curves are effective for binary classification, extending them to multi-class problems can be challenging and finally, it assumes independence of observations.
